# ENGINES: exploring single nucleotide variation in entire human genomes

**DOI:** 10.1186/1471-2105-12-105

**Published:** 2011-04-19

**Authors:** Jorge Amigo, Antonio Salas, Christopher Phillips

**Affiliations:** 1 Grupo de Medicina Xenómica, CIBERER, Universidade de Santiago de Compostela, Santiago de Compostela, Galicia, Spain; 2 Unidade de Xenética Forense, Instituto de Medicina Legal, Facultade de Medicina, Universidade de Santiago de Compostela. Santiago de Compostela, Galicia, Spain

## Abstract

**Background:**

Next generation ultra-sequencing technologies are starting to produce extensive quantities of data from entire human genome or exome sequences, and therefore new software is needed to present and analyse this vast amount of information. The 1000 Genomes project has recently released raw data for 629 complete genomes representing several human populations through their Phase I interim analysis and, although there are certain public tools available that allow exploration of these genomes, to date there is no tool that permits comprehensive population analysis of the variation catalogued by such data.

**Description:**

We have developed a genetic variant site explorer able to retrieve data for Single Nucleotide Variation (SNVs), population by population, from entire genomes without compromising future scalability and agility. ENGINES (ENtire Genome INterface for Exploring SNVs) uses data from the 1000 Genomes Phase I to demonstrate its capacity to handle large amounts of genetic variation (>7.3 billion genotypes and 28 million SNVs), as well as deriving summary statistics of interest for medical and population genetics applications. The whole dataset is pre-processed and summarized into a data mart accessible through a web interface. The query system allows the combination and comparison of each available population sample, while searching by rs-number list, chromosome region, or genes of interest. Frequency and F_ST _filters are available to further refine queries, while results can be visually compared with other large-scale Single Nucleotide Polymorphism (SNP) repositories such as HapMap or Perlegen.

**Conclusions:**

ENGINES is capable of accessing large-scale variation data repositories in a fast and comprehensive manner. It allows quick browsing of whole genome variation, while providing statistical information for each variant site such as allele frequency, heterozygosity or F_ST _values for genetic differentiation. Access to the data mart generating scripts and to the web interface is granted from http://spsmart.cesga.es/engines.php

## Background

The appearance of large-scale online compilations of human variation has profoundly changed the population genetics field in the last decade. Private companies such as Perlegen Sciences [1], global collaborations such as HapMap [2] and high density Single Nucleotide Polymorphism (SNP) genotyping of the CEPH human genome diversity panel by groups from the Universities of Stanford [3] and Michigan, have provided extensive variation catalogues for geneticists to examine differences amongst a wide range of human populations. But although most genome studies have released their raw data to the public there has been a lack of web interfaces that allow population genetics based interpretation of the data. Indeed, in the current era of rapidly expanding numbers of publicly released complete human sequences there is an evident need to develop online data browsers that can collate and represent portions of the data relevant for particular fields of research.

The 1000 Genomes project http://www.1000genomes.org/ is a public initiative that aims to collect a very large proportion of variation detectable by next generation sequencing techniques of human genomes from several worldwide populations. The first pilot study (Pilot 1) assessed the strategy of sharing data across samples on whole genome sequencing results with relatively low coverage (2-4x). It presented 179 genomes from the four different population panels previously characterised by HapMap (CEU, CHB, JPT and YRI) describing ~14 million variants. The recent release of an interim analysis of the project's Phase I has considerably enriched the data available: 629 entire genomes from 12 different populations, describing ~28 million variants. These populations are: individuals of African ancestry in Southwest USA (ASW), Utah residents with N & W European ancestry from the CEPH collection (CEU), Han Chinese in Beijing, China (CHB), Han Chinese South (CHS), Finnish in Finland (FIN), British in England and Scotland (GBR), Japanese in Tokyo, Japan (JPT), Luhya in Webuye, Kenya (LWK), individuals of Mexican ancestry in Los Angeles, California (MXL), Puerto Ricans in Puerto Rico (PUR), Tuscans in Italy (TSI), and Yoruba in Ibadan, Nigeria (YRI).

Although the 1000 Genomes project has already started to release results there are few publicly available bioinformatics tools that allow thorough exploration of such data. The Integrative Genomics Viewer http://www.broadinstitute.org/igv/home is a Java-based desktop application that permits visual browsing of the 1000 Genomes Pilot 1, 2, and 3 calls (among other tracks). Alternatively the 1000 Genomes Browser http://browser.1000genomes.org/ is a web tool that permits visualization of the variant sites against the reference sequence, and dynamic loading of tracks of interest (functional consequence, conservation, etc.). The latter provides a very simple and intuitive way to browse the 1000 Genomes results, but it does not provide basic variation statistics for population studies such as allele frequency or genetic differentiation of the genomes included in the project. More importantly, the 1000 Genomes Browser reviews the sequence surrounding just a single query at a time whether variant site, gene or chromosome segment. Furthermore, the 1000 Genomes browser is currently confined to the six Pilot 2 sequences.

## Construction and content

We have developed a human genome variant site browser: ENGINES dedicated, in the first instance, to the flexible and thorough analysis of the Single Nucleotide Variation (SNV) catalogue generated from the 1000 Genomes Phase I interim analysis, although it will subsequently integrate new whole genome sequence data from other sources as this becomes publicly available.

### Design and capabilities

**Figure 1:**
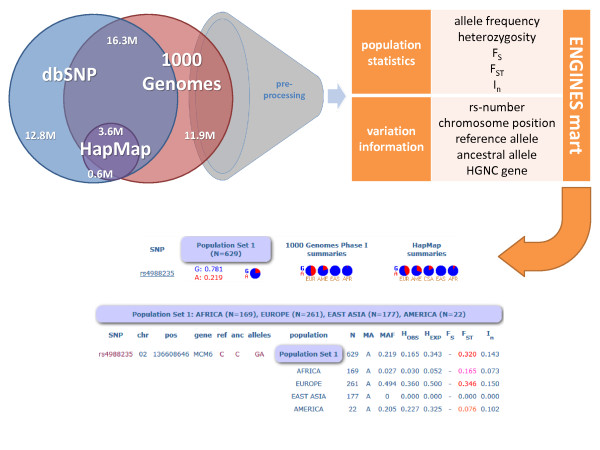
**Data workflow**. Pre-processing of large-scale human variation sources, creation of a data mart from population and variation specific data plus display of results through the web interface. The information taken from dbSNP is used just for mapping purposes - full content is not present on the data mart. HapMap release 28 describes 4,166,638 SNPs all listed by dbSNP build 132, 3,654,377 of these are present in 1000 Genomes Phase I. A total of 28,210,483 unique variants have been detected by the 1000 Genomes Phase I interim analysis, 16,313,540 already listed in dbSNP build 132 (which currently comprises 29,133,600 SNPs in total). Screenshots show a single SNP search for rs4988235; this SNP is located in the *MCM6 *gene but influences the lactase gene (*LCT*); the intercontinental global F_ST _value is higher than expected (highlighted in red; 0.320) as it corresponds to the locus that shows the strongest signal of positive selection in the human genome.

As shown in Table 1 the volume of data is already very large, and with the goal to aggregate all available new whole genome data, summarizing approaches are essential to allow easy data management and to perform quick non-batched queries [4]. The whole dataset is pre-processed using a pipeline of customized PERL scripts and collated into a seven gigabyte MySQL data mart, containing only the summarized statistics, arranged by population, including allele frequencies, heterozygosity or minor allele frequency (MAF). This data mart is then queried through a PHP web interface with the main aim of permitting multiple SNV queries of entire genomes with a single step, dictated by user-defined nucleotide range, HGNC gene symbol list or rs-number list applied to the user's selection from different global population panels (Figure 1).

**Table 1:** Data mart facts

1000 Genomes Phase I			
Populations	*N *genomes	Variant Sites*^1^	Variant Genotypes
ASW	24	14,037,711	336,905,064
CEU	90	10,983,038	988,473,420
CHB	68	9,490,259	645,337,612
CHS	25	7,588,537	189,713,425
FIN	36	8,680,985	312,515,460
GBR	43	9,376,836	403,203,948
JPT	84	10,071,464	846,002,976
LWK	67	17,279,531	1,157,728,577
MXL	17	8,513,411	144,727,987
PUR	5	6,354,128	31,770,640
TSI	92	11,368,655	1,045,916,260
YRI	78	16,567,193	1,292,241,054
TOTAL	629	28,210,483	7,394,536,423
			
**HapMap release 28**			

Populations	*N *samples	Variant Sites	Variant Genotypes
ASW	53	1,543,440	81,802,320
CEU	121	2,816,160	340,755,360
CHB	139	2,635,473	366,330,747
CHD	109	1,312,139	143,023,151
GIH	101	1,409,285	142,337,785
JPT	116	2,561,639	297,150,124
LWK	110	1,527,108	167,981,880
MEX	58	1,453,424	84,298,592
MKK	156	1,532,287	239,036,772
TSI	102	1,420,285	144,869,070
YRI	153	3,151,427	482,168,331
TOTAL	1218	4,170,392	2,489,754,132

The comparison of all the variability information present on the 1000 Genomes Phase I with HapMap release 28 indicates that although HapMap doubles the sample size, 1000 Genomes triples the number of genotypes due to the superior density of variants (this is particularly interesting in the YRI population which is now even more completely described than before). The number of non-monomorphic sites is reported as "variant sites".

*^1^Variant sites refer to the number of bi-allelic markers observed in each population group. Note that the Phase I does not contain information on tri- or tetra-allelic variants while in Pilot 1 there are more than 16,000 tri-allelic SNVs plus 12 tetra-allelic SNVs (data not shown).

The statistics tab displays a table describing each variation result in columns: variation code, chromosome, chromosome position, gene, reference allele (from the current human reference genome GRCh37), ancestral allele (from the Chimpanzee genome), alleles found in all present genotypes, populations queried, number of samples (N), the minor allele (MA) and its frequency (MAF), observed and expected heterozygosities (H_OBS _and H_EXP_), local inbreeding (F_S_), genetic differentiation (F_ST_, which is presented on different colours depending on meaning steps: under 0.05, 0.15, 0.25 and above 0.25) and informativeness of population group assignment (I_n_). In ENGINES the emphasis is on multiple queries as a flexible, and in terms of genome portions that can be queried, broader alternative to the single marker queries offered by e.g. the 1000 Genomes browser.

Rapid responses to queries of dense genomic data have been engineered into the browser by use of pre-calculated SNV allele frequencies based on population groupings, an approach already successfully implemented in the population-based SNP frequency browser: SPSmart [5]. ENGINES therefore exploits one of the major assets of the 1000 Genomes Pilot 1 data, the improved detection and characterization of low frequency nucleotide variation, whether defined by population, genome position or overall MAF with linked references made at the same time to existing data in dbSNP or HapMap. For example, ENGINES can be used to search in batch mode for:

1) SNVs in specific genes or gene families;

2) SNVs at varying frequencies in different global population panels;

3) Novel variants or SNVs at very low MAF, which are now adequately catalogued and validated; For any selected SNV set, ENGINES can also calculate a range of statistical indices of interest for human population genetics studies.

### Maintaining the data mart

The update frequency of the databases currently accessed by ENGINES varies considerably. Thus, while dbSNP is expected to release updates on a yearly basis, having been updated once or twice a year since 2004, Phase I is a static resource, and the project's final data releasing policy has not been publicly stated. The data mart will be updated with the 1000 Genomes final variant data upon release, in addition relevant whole genome sequencing data in the public domain from other initiatives will also be collated and included.

Originally, ENGINES used 1000 Genomes' Pilot 1 as an appropriate testing dataset. It was mapped to the old NCBI36/hg18 human genome reference, and for that reason we were forced to use dbSNP build 130 as the most up to date standard for describing all variants when possible. When the 1000 Genomes project released this Phase I interim analysis we decided to update our tool to a more appropriate testing dataset, implying adapting the data parsing scripts and upgrading the mapping reference to the new GRCh37/hg19. This later fact allowed ENGINES to update the variants description reference to dbSNP build 132, and considering that human reference versions tend to be fixed for a long time this should allow the internal data marts to be easily updated when new data is released, either from the number of genotypes side (new projects or existing projects update) or either from the variants description point of view (dbSNP updates, which occur approximately once a year).

The most common population genetics statistical indices have been implemented and summarized in the ENGINES data mart, but other metrics of interest could be easily implemented with just the raw data pre-processing script requiring updates: equivalent to two computing days due to the flexibility of the pipeline developed. In fact, and although it took ENGINES 1 month to be adapted to the new 1000 Genomes Phase I interim analysis data release policy, updating the data mart with the whole project's final data would take only 1 week even considering that the number of genomes is expected to be multiplied by 5.

## Utility and discussion

Since several alternative means are available for researchers to access 1000 Genomes SNP data it is important to outline the advantages offered by the ENGINES browser in comparison to other approaches, which we see as complementary in their output, rather than competing to provide the same type of data. ENGINES is primarily designed to serve population genetics studies and therefore has several key features built in:

1. A straightforward system to download the individual genotypes for the SNPs, genes and populations queried. This permits direct input into population analysis algorithms such as *Structure *[6] or *Arlequin *[7].

2. Each database, population and SNV can be visually compared side by side, and the relevant data for SNVs and populations can be downloaded in one session from each database query.

3. F_ST _values, amongst other metrics, can be collated for the entire genome-wide or exome SNV catalogue.

4. Lists of SNPs or genes are easily handled offering a more rapid and straightforward system than the SNP by SNP queries of the 1000 Genomes browser.

5. Genotyping coverage can be assessed at a glance by reviewing which SNPs and databases show incomplete genotyping.

6. Different filters are available that allow the selective listing of sets of variants according to different thresholds defined by the user (e.g. F_ST _, MAF, etc).

ENGINES processed more than 7.3 billion genotypes and ~28 million unique variants in the Phase I interim analysis of the 1000 Genomes project (Table 1), of which 11.9 million were not previously described in dbSNP 132 (Figure 1). To illustrate the ease with which the ENGINES browser can add extra data to existing genome-wide analyses, of relevance for population genetics studies, we collated the total variant number by population group (Table 1). As expected from the demographic history of human populations, ENGINES clearly indicates the two sub-Saharan samples (LWK and YRI) contain more variants than any other population or set of populations, followed by the African-American sample (ASW). The data in this population break-down is different to the one provided by the 1000 Genomes analysis [8] because the latter targeted low coverage analysis of only the CEU, YRI, CHB, and JPT (Pilot 1) or exon regions (Pilot 3). Our data reveals interesting differences of SNP density that could contribute to the study of global patterns of natural selection (Table 1).

F_ST _is a metric of genetic differentiation [9] between populations. It is also well known that the action of natural selection can locally cause systematic deviation in F_ST _values for a selected gene and nearby markers. Thus, when compared with the action of a neutral evolving gene, high F_ST _values might signal the action of local directional selection, while a decrease of F_ST _values would be suggestive of balancing selection. Analysis of F_ST _values on a genome-wide scale has already been demonstrated to be very useful for mapping genes under selection [10]. The 1000 Genomes pilot project has allowed the calculation of F_ST _values for the first time in the framework of a whole genome sequencing project [8], and has already revealed preliminary features relating to new regions that could have been subject to natural selection. In a step forward, ENGINES provides F_ST _values for different population or continental combinations selected by the user and centred on the most current data release of 1000 Genomes. Access to this information is straightforward, and genotypes can be easily downloaded *ad hoc *for the regions of interest in order to carry out further analyses. By way of example, additional file 1 provides a snapshot of genome-wide F_ST _values when considering a four-way inter-continental comparison (Africa, Europe, Asia, and America). Additional file 2 records the top F_ST _values (>0.9) plotted in Figure S1, indicating that a large proportion of these values fall within known genes but notably a significant proportion are also located in uncharacterized genomic regions; therefore, providing new targets of considerable interest for further evolutionary and population genetic research. In addition, analysis of populations to a more extended intra-continental scale allows a refinement in the ability to search at greater population depth signals of localized adaptation.

Finally, an indirect assessment of the quality of ENGINES can be undertaken by the user by comparing SNP frequencies in Phase I with those of HapMap for the overlapping SNPs and populations (CEU, CHB, JPT, and YRI). Minor differences or discrepancies are possible but can be attributed to missing data or potential genotyping errors (due e.g. to Phase I SNV detection based on ultra-sequencing at low coverage). We have indeed observed genotyping discrepancies between genotypes reported in HapMap and those reported in Phase I for the same samples (data not shown).

## Conclusions

ENGINES is capable of accessing large variation data repositories in a fast and comprehensive manner. We have shown that 1000 Genomes variant data, which represents the largest current whole human genome variation repository, is easily summarized and queried by ENGINES with a straightforward yet thorough approach for handling multiple sites across multiple genomes. ENGINES allows fast and easy browsing of whole genome variation by using a simple and intuitive web interface that performs queries in seconds and displays results in an efficient manner, while providing statistical information of each variation site such as frequency, heterozygosity or genetic differentiation among populations that are already pre-calculated and presented on demand.

## Availability

The data mart generating scripts are a set of Perl files that are freely available on the software section of ENGINES. Access to these scripts and to the main web interface is granted from http://spsmart.cesga.es/engines.php

## Authors' contributions

JA carried out the design, programming and implementation of the software, and drafted the manuscript. AS and CP participated in the design of the software, and helped to draft the manuscript. All authors read and approved the final manuscript.

## Supplementary Material

Additional file 1 **Figure S1 - Genome-wide F_ST _values**. Chromosome position in Mb is given in the X-axis, and F_ST _values are plotted on the Y-axis. F_ST _values are shown in black or red (red shows values that are exceptionally high: corresponding to the upper 2.5% of the empirical distribution of F_ST _values). The yellow line shows the average of F_ST _values for non-overlapping genomic windows of 1 Mb. Gaps correspond to heterochromatic staining regions near centromeres. Click here for file

Additional file 2 **Table S1 - Top F_ST _values**. List of SNVs showing the top F_ST _values (above 0.9) for the four main continental group and their pairwise combinations (AFR = Africa; EAS = East Asia; EUR = Europe, and AME = America). Genes and rs-numbers are provided when available. Click here for file
