# Supplementary figures and images for: ENGINES: exploring single nucleotide variation in entire human genomes
Source: BMC Bioinformatics. 2011 Apr 19;12:105. doi: 10.1186/1471-2105-12-105 (PMC3107182; doi:10.1186/1471-2105-12-105)

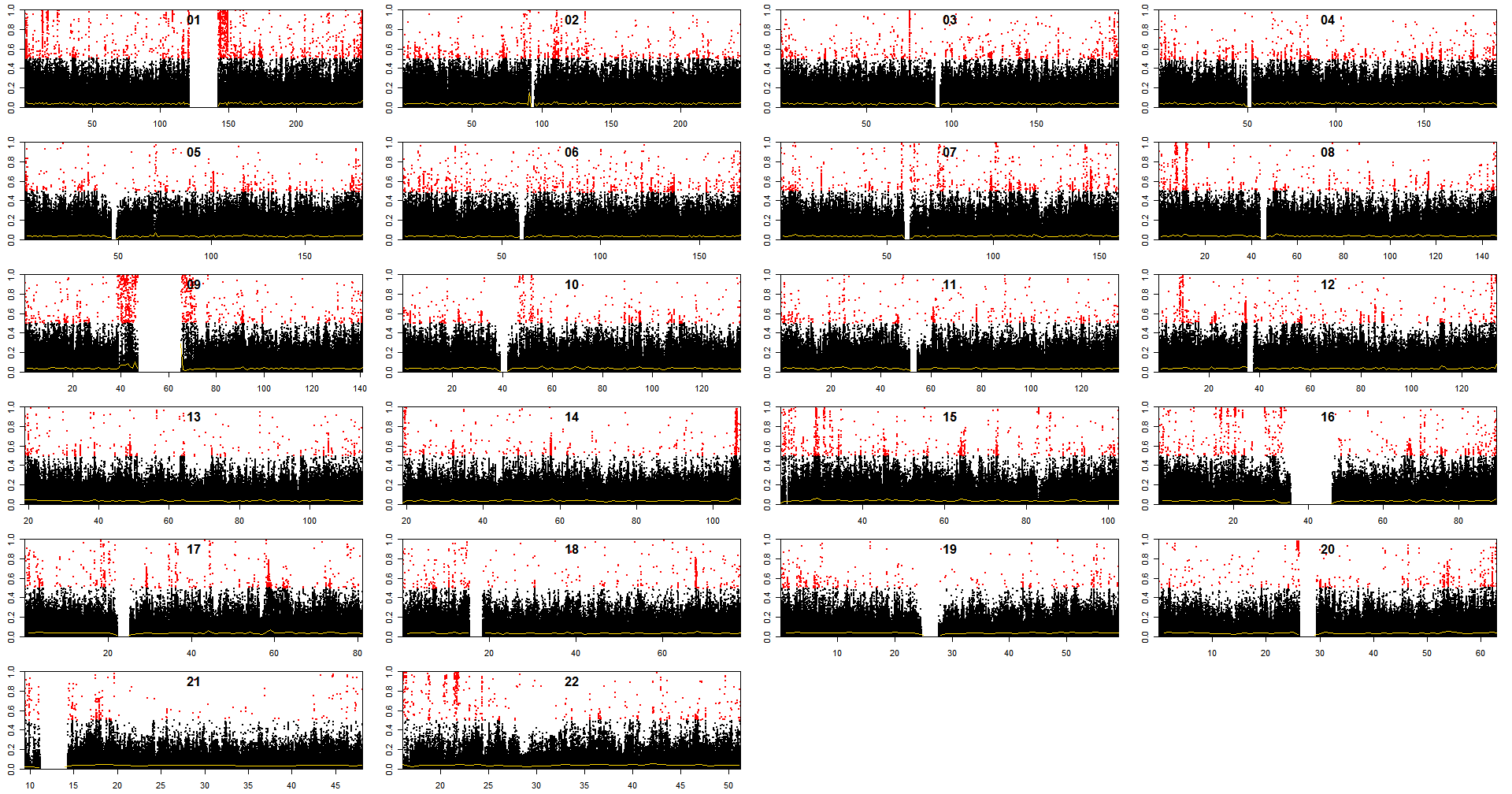

Supplement: Additional file 1 — Figure S1 - Genome-wide FST values. Chromosome position in Mb is given in the X-axis, and FST values are plotted on the Y-axis. FST values are shown in black or red (red shows values that are exceptionally high: corresponding to the upper 2.5% of the empirical distribution of FST values). The yellow line shows the average of FST values for non-overlapping genomic windows of 1 Mb. Gaps correspond to heterochromatic staining regions near centromeres. [file 1471-2105-12-105-S1.PNG]
